# What is the impact of the use of transanastomotic feeding tube on patients with esophageal atresia: a systematic review and meta-analysis

**DOI:** 10.1186/s12887-018-1359-5

**Published:** 2018-12-12

**Authors:** Chuan Wang, Liwei Feng, Yanan Li, Yi Ji

**Affiliations:** 1 Department of Pediatric Surgery, West China Hospital of Sichuan University, Chengdu, 610041 China; 2 Department of Pediatric Surgery, West China Hospital of Sichuan University, Chengdu, 610041 China; 3 Department of Pediatric Surgery, West China Hospital of Sichuan University, Chengdu, 610041 China; 4 Department of Pediatric Surgery, West China Hospital of Sichuan University, #37 Guo-Xue-Xiang, Chengdu, 610041 China

**Keywords:** Esophageal atresia, Tracheoesophageal fistula, Transanastomotic feeding tube, Complication, Stricture

## Abstract

**Background:**

The transanastomotic feeding tube (TAFT) is widely used around the world in patients with esophageal atresia (EA). However, the safety of the use of TAFT is still unknown and remains to be clarified.

**Methods:**

The following electronic databases were searched: PubMed, EMBASE and Cochrane. Studies comparing outcomes in patients with the use of TAFT (TAFT+) and patients without the use of TAFT (TAFT-) were scrutinized. The quality of included studies was evaluated with the Newcastle–Ottawa scale score. Statistical heterogeneity was assessed using the *I*^2^ value. A fixed or random-effect model was applied.

**Results:**

Four retrospective controlled studies involving 455 patients were included. The pooled estimates showed that the use of TAFT significantly increased the risk of stricture, with a risk ratio (RR) of 1.83 (95% CI 1.30–2.58; *P* = 0.0005). The meta-analyses of other postoperative complications did not show significant differences between TAFT+ and TAFT- group, with a RR of 1.65 (95% CI 0.93–2.93; *P* = 0.09) for anastomotic leakage, 0.91 (95% CI 0.34–2.44; *P* = 0.85) for sepsis, 1.89 (95% CI 0.22–16.20; *P* = 0.56) for tracheomalacia, 0.50 (95% CI 0.13–1.93; *P* = 0.31) for gastroesophageal reflux, 1.29 (95% CI 0.28–5.92; *P* = 0.74) for wound infection, and 0.97 (95% CI 0.03–36.75; *p* = 0.99) for pneumonia.

**Conclusions:**

This study demonstrates that the use of TAFT in patients with EA significantly increases the risk of stricture. However, TAFT is not associated with other complications, including anastomotic leakage, sepsis, tracheomalacia, gastroesophageal reflux, wound infection and pneumonia.

## Background

Esophageal atresia (EA) is a rare congenital gastrointestinal anomaly that affects 1 per 4000 newborns [1–3]. Approximately 93% of EA are associated with tracheoesophageal fistula [1]. Although the survival rate of EA is higher than 90% with the advances in perioperative management and surgical techniques, the postoperative complications are still frequent [4–6]. The most common complication is stricture with an estimated prevalence of 40%, followed by anastomotic leakage occurring in about 20% of patients [6, 7].

In 1996, Moriarty et.al [8] first reported the use of transanastomotic feeding tube (TAFT) in patients with EA. Currently, TAFT is widely used around the world [9]. However, studies investigating the effects of TAFT on patients with EA have conflicting results [10–14]. Proponents recommend that TAFT is able to allow earlier initiation of enteral feeds and potentially supports anastomosis as stenting [8, 10]. However, other researchers believe that TAFT is implicated in increased risk of stricture and anastomotic leakage [11, 13, 14]. Unfortunately, most studies had small sample sizes. The benefits and risks of TAFT in patients with EA still remain to be clarified.

Thus, we conducted this meta-analysis with the aim to elucidate the safety of TAFT in patients with EA by evaluating the prevalence of postoperative complications.

## Methods

### Study selection

Only controlled studies comparing outcomes in patients with the use of TAFT (TAFT+) and without the use of TAFT (TAFT-) were eligible for inclusion. In addition, eligible studies were requested to report at least one of the following complications: stricture and anastomotic leakage. The eligible literatures were limited to being published in English.

### Search strategy

Two investigators (C.W. and L.F.) systematically searched the PubMed, EMBASE and Cochrane Library databases to identify studies and determine eligibility. The core search terms were ‘esophageal’, ‘oesophageal’, ‘atresia’, ‘tracheoesophageal fistula’, ‘transanastomotic’, ‘transanastomosis’ and ‘tube’, and these words were combined with Boolean operators AND, OR, and NOT. Both of the two reviewer scrutinized titles and abstracts, and screened full-text manuscripts of selected studies eligible for inclusion criteria independently. Reference lists of eligible literatures were scrutinized to identify any other potential studies.

### Data extraction and quality assessment

We defined anastomotic leakage and stricture as the primary outcomes. Other complications, including sepsis, tracheomalacia, gastroesophageal reflux, wound infection and pneumonia, were defined as the secondary outcomes. Data, including first author, publication year, numbers of cases and controls, study design, characteristics of the study population, the primary outcomes, and the secondary outcomes, were extracted using a standardized data-extraction sheet by two reviewers (C.W. and L.F.). Disagreements were resolved by checking the manuscripts and/or contacting the authors if necessary. The quality of included cohort studies was assessed in accord with the Newcastle-Ottawacriteria scale (NOS) scores [15]. The total scores were ranged from 0 to 9 for cohort studies. Studies with a score of at least 6 were categorized as “high quality.”

### Statistical analysis and exploration of heterogeneity

All statistical analyses were conducted by using Reviewer Manager 5.3 (Cochrane Collaboration). Pooled results were expressed as the risk ratio (RR) with 95% confidence intervals (CIs) for all end points. The Mantel–Haenszel method was used to combine the summary statistics. The funnel plots were used to assess the potential for publication bias. The *I*^2^ method was used to assess heterogeneity among studies, with higher *I*^2^ value indicating higher heterogeneity. If the *I*^2^ value was less than 50%, a fixed-effects model of analysis was used. Otherwise, a random-effects model was used.

## Results

**Fig. 1:**
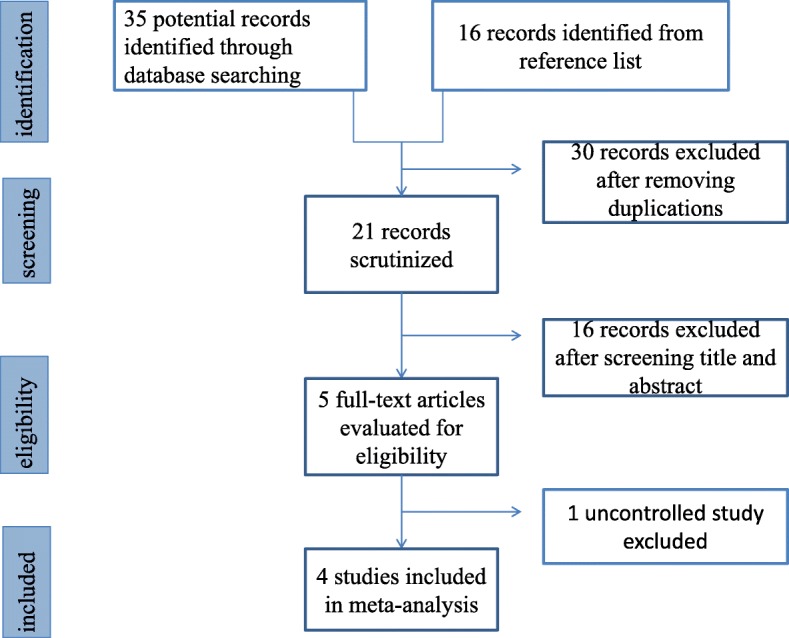
Flow chart of study selection

In total, we identified 51 articles through online search and reference lists of relevant publications (Fig. 1). After scrutinizing the titles and abstracts, a total of five full-text manuscripts were assessed for eligibility. Finally, four of five studies met the inclusion criteria. All of these studies were retrospective observational clinical studies [10–13]. The characteristics of the four studies were shown in Table 1. A total of 455 patients with EA were assigned to the TAFT+ group (*n* = 335) or the TAFT- group (*n* = 120). Data regarding outcomes of each study are summarized in Table 2. No obvious publication bias was detected in all analyses.

**Table 1:** Characteristics of included studies

Study	Study type	Sample size	Age at surgery (day)	Gestational age (week)	Birth weight (kg)	weight (kg)	NOS
Alabbad SI 2009	OCS (retrospective)	TAFT+:9	NA	39.00 ± 2.1	3.13 ± 0.55	NA	7
		TAFT-:11	NA	37.64 ± 2.5	2.82 ± 0.69	NA	
Fusco JC 2017	OCS (retrospective)	TAFT+:81	2.4	NA	NA	2.69	7
		TAFT-:29	2.3	NA	NA	2.71	
Narayanan SK 2017	OCS (retrospective)	TAFT+:14	NA	35.64 ± 2.60	2.30 ± 0.23	NA	7
		TAFT-:19	NA	36.52 ± 2.20	2.50 ± 0.32	NA	
Lal DR 2018	OCS (retrospective)	TAFT+:231	NA	NA	NA	NA	6
		TAFT-:61	NA	NA	NA	NA	

*TAFT* ransanastomotic feeding tube, *OCS* observational clinical study, *NOS* Newcastle-Ottawa scale, *NA* not available

**Table 2:** Summary of the outcomes of included studies

Study	Sample size	Anastomotic leakage	Stricture	Sepsis	Tracheomalacia	Gastroesophageal reflux	Wound infection	Pneumonia
Alabbad SI 2009	TAFT+:9	2 (22%)	2 (22%)	3 (33%)	1 (11%)	0 (0%)	2 (22%)	0 (0%)
	TAFT-:11	1 (8%)	4 (36%)	4 (36%)	2 (18%)	3 (27%)	1 (8%)	4 (36%)
Fusco JC 2017	TAFT+:81	12 (15%)	45 (56%)	NA	NA	NA	NA	NA
	TAFT-:29	2 (7%)	5 (38%)	NA	NA	NA	NA	NA
Narayanan SK 2017	TAFT+:14	2 (14%)	4 (29%)	2 (14%)	4 (29%)	2 (14%)	1 (7%)	7 (50%)
	TAFT-:19	2 (11%)	3 (16%)	3 (16%)	1 (5%)	3 (16%)	2 (11%)	2 (11%)
Lal DR 2018	TAFT+:231	111 (48%)	46 (20%)	NA	NA	NA	NA	NA
	TAFT-:61	18 (30%)	8 (13%)	NA	NA	NA	NA	NA

*TAFT* transanastomotic feeding tube *NA* not available

### Primary outcome

#### Anastomotic leakage

**Fig. 2:**
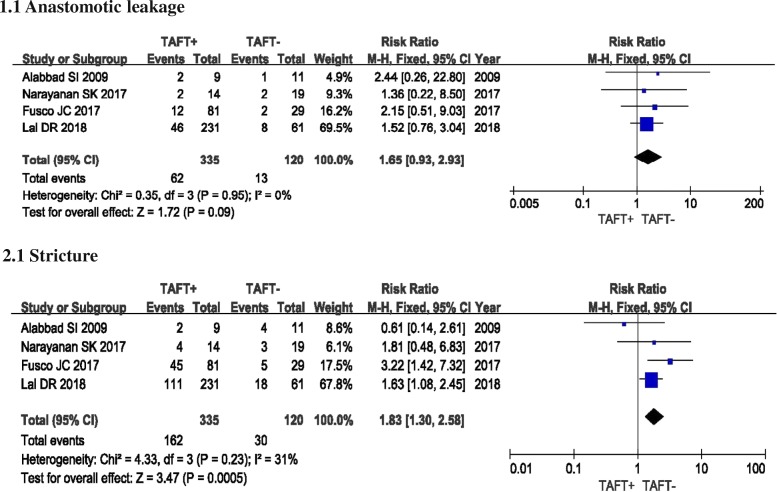
Forest Plot Showing Risk Ratio (RR) in occurrence rate of anastomotic leakage and stricture in the transanastomotic feeding tube (TAFT) + vs TAFT- Groups

All four studies investigated the postoperative occurrence of anastomotic leakage in patients with or without the use of TAFT [10–13]. The rate of anastomotic leakage was 18.5% (62, *n* = 335) in TAFT+ group and 10.8% (13, *n* = 120) in TAFT- group. There was no significant heterogeneity among studies (*I*^2^ = 0%). The overall pooled RR was 1.65 (95% CI 0.93–2.93; *P* = 0.09). The result showed no significant discrepancy for anastomotic leakage between two groups (Fig. 2).

### Stricture

Stricture was reported in all four studies [10–13]. Two studies reported that the use of TAFT was associated with a high rate of stricture [11, 13]. In total, there were 162 of 335 patients in TAFT+ group and 30 of 120 patients in TAFT- group diagnosed as stricture (Fig. 2). The *I*^2^ method identified low heterogeneity among four studies (*I*^2^ = 31%). The pooled estimate showed the use of TAFT significantly increased the risk of stricture, with a RR of 1.83 (95% CI 1.30–2.58; *P* = 0.0005).

### Secondary outcome

#### Sepsis

**Fig. 3:**
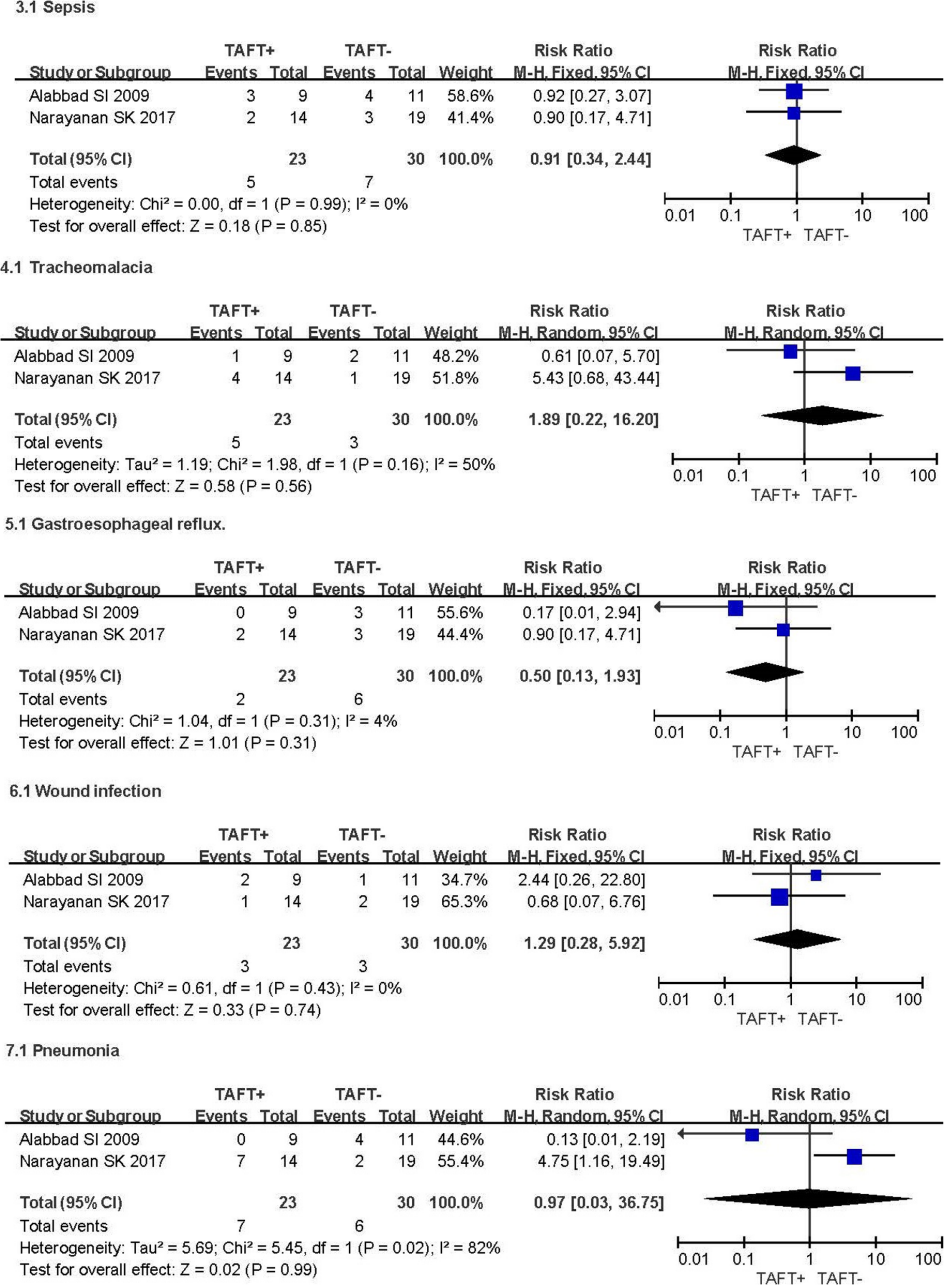
Forest Plot Showing Risk Ratio (RR) in occurrence rates of other complications in the TAFT+ vs TAFT- Groups

Two studies investigated the presence of sepsis. A total of 53 patients were involved [10, 12]. No discernible heterogeneity was detected with *I*^2^ = 0% (Fig. 3). There was no significant discrepancy for sepsis between TAFT+ and TAFT- group, with a RR of 0.91(95% CI 0.34–2.44; *P* = 0.85).

### Tracheomalacia

Two studies recorded the occurrence of tracheomalacia [10, 12]. A moderate heterogeneity was examined with *I*^2^ = 50%. A random-effects model of analysis was used to calculate the pooled RR. No discernible difference for tracheomalcia was detected between TAFT+ and TAFT- groups, with a RR of 1.89 (95% CI 0.22–16.20; *P* = 0.56) (Fig. 3).

### Gastroesophageal reflux

Gastroesophageal reflux was recorded in two studies [10, 12]. The pooled estimate indicated no significant difference between TAFT+ and TAFT- groups, with a RR of 0.50(95% CI 0.13–1.93; *P* = 0.31; *I*^2^ = 4%) (Fig. 3).

### Wound infection

Wound infection as an outcome was reported in two studies, with a total of 53 patients involved [10, 12]. No heterogeneity was detected between two studies (*I*^2^ = 0%). The occurrence of wound infection was not significantly different between two groups, with a RR of 1.29 (95% CI 0.28–5.92; *P* = 0.74) (Fig. 3).

### Pneumonia

Pneumonia after operation was reported in two studies [10, 12]. There were 23 patients and 30 patients in TAFT+ group and TAFT- group, respectively. A random-effects model of analysis was used owing to high heterogeneity (*I*^2^ = 82%). The occurrence of pneumonia was not significantly different between two groups, with a RR of 0.97 (95% CI 0.03–36.75; *P* = 0.99) (Fig. 3).

## Discussion

EA is a rare malformation. The operation for EA is inevitable. However, the perioperative management for EA is variable [1]. Clinically, TAFT is widely used to initiate feeds. Dave Lal et al. [16] performed an international survey involving 170 pediatric surgeons from 31 countries. The results revealed that 83% of surgeons placed TAFT. Another study indicated that 90% of 168 surgeons used TAFT [13]. Although TAFT is widely used, the advantages and disadvantages are still debated. The important benefits of TAFT include early enteral feeding and reduction of total parenteral nutrition duration. However, there was no significant difference in the median number of postoperative days starting enteral feeds and total parenteral nutrition duration between TAFT+ and TAFT- group in retrospective studies [10, 13]. Some studies suggested the TAFT was related to harm. Little evidence exists regarding the safety of TAFT in patients with EA. The concern needs to be delineated.

Postoperative complications occur in 62% patients with EA [7, 13]. Anastomotic leakage and stricture occur frequently in approximately 20 and 40% of the population, respectively. Our study indicates that the use of TAFT is not associated with a higher risk of anastomotic leakage. Lal DR et al. suggested that TAFT might be associated with stricture [13]. Unfortunately, robust evidence is lacking to confirm the risk. Our meta-analysis confirmed that the use of TAFT was associated with an increased risk of stricture. This result is consistent with another study showing that the use of TAFT is associated with an increase of stricture and less ananstomotic collagen formation in animal models [17]. Two potential mechanisms were raised to explain the impact of TAFT on the occurrence of stricture, including mechanical shearing at the anastomosis and dilation of the lower esophageal sphincter resulting in increased exposure of the anastomosis to reflux [13]. It is high time to reconsider whether the worldwide use of TAFT is a right perioperative management in patients with EA.

There is a hypothesis that TAFT might dilate the esophageal sphincter, and therefore result in exposure of the anastomosis to reflux. It is concerned whether the use of TAFT can increase the risk of gastroesophageal reflux. Gastroesophageal reflux may lead to early postoperative complications including stricture formation, aspiration pneumonia and failure to thrive, or result in late complications, such as Barrett’s esophagus and cancer [18–20]. In the present study, however, we found that the utilization of TAFT in patients with EA was not related to the development of gastroesophageal reflux. Thus, the hypothesis that TAFT leads to exposure of the anastomosis to reflux in patients with EA might be untenable. Additionally, our meta-analysis revealed that TAFT did not increase the risk of pneumonia, suggesting that there might have no relationship between TAFT and gastroesophageal reflux. The risks of other complications using TAFT, including sepsis, tracheomalacia and wound infection, were also assessed. Similarly, we demonstrated that the use of TAFT did not significantly increase the risk of these complications.

Some limitations of this study should be recognized. Although all four studies were of high quality in accordance with the Newcastle-Ottawa criteria (all NOS ≥ 6), these studies were retrospective control studies. In addition, two of four studies had small sample sizes.

## Conclusions

This meta-analysis provides valuable evidence regarding the risks of postoperative complications of the use of TAFT in patients with EA. Our study reveals that the use of TAFT significantly increases the risk of stricture. In addition, our data demonstrate that the use of TAFT is not associated with other complications, including anastomotic leakage, sepsis, tracheomalacia, gastroesophageal reflux, wound infection and pneumonia. Future prospective, randomized, and controlled studies are needed to extend these conclusions toward to further confirm the benefits and risks of the use of TFAT in patients with EA.

## Abbreviations

CI: Confidence interval
EA: Esophageal atresia
NOS: Newcastle-Ottawacriteria scale
RR: Risk ratio
TAFT: Transanastomotic feeding tube
